# Opportunities for Improved Disease Surveillance and Control by Use of Integrated Data on Animal and Human Health

**DOI:** 10.3389/fvets.2019.00301

**Published:** 2019-09-13

**Authors:** Hans Houe, Søren Saxmose Nielsen, Liza Rosenbaum Nielsen, Steen Ethelberg, Kåre Mølbak

**Affiliations:** ^1^Department of Veterinary and Animal Sciences, University of Copenhagen, Copenhagen, Denmark; ^2^Department of Infectious Disease Epidemiology and Prevention, Statens Serum Institut, Copenhagen, Denmark; ^3^Global Health Section, Department of Public Health, University of Copenhagen, Copenhagen, Denmark

**Keywords:** one health (OH), animal health, human health, databases, Denmark

## Abstract

The global challenges and threats from infectious diseases including antimicrobial drug resistance and emerging infections due to the rapidly changing climate require that we continuously revisit the fitness of our infrastructure. The databases used for surveillance represent an important infrastructure. Historically, many databases have evolved from different needs and from different organizations. Despite growing data storage and computing capacities, data are, however, rarely used to their full potential. The objective of this review was to outline different data sources available in Denmark. We applied a one-health perspective and included data sources on animal demographics and movements, medicine prescription, diagnostic test results as well as relevant data on human health. Another objective was to suggest approaches for fit-for-purpose integration of data as a resource for risk assessment and generation of evidence for policies to protect animal and human health. Danish databases were reviewed according to a systematic procedure including ownership, intended purposes of the database, target and study populations, metrics and information used, measuring methods (observers, diagnostic tests), recording procedures, data flow, database structure, and control procedures to ensure data quality. Thereby, structural metadata were gathered across available Danish databases including animal health, zoonotic infections, antimicrobial use, and relevant administrative data that can support the overall aim of supporting risk assessment and development of evidence. Then illustrative cases were used to assess how combinations and integration of databases could improve existing evidence to support decisions in animal health policies (e.g., combination of information on diseases in different herds or regions with information on isolation of pathogens from humans). Due to the complexity of databases, full integration at the individual level is often not possible. Still, integration of data at a higher level (e.g., municipality or region) can provide important information on risks and hence risk management. We conclude by discussing how databases by linkage can be improved in the future, and emphasize that legal issues are important to address in order to optimize the use of the available data.

## Introduction

The growing possibilities for collecting information on demographic factors on animals and humans, their environment and movements, disease and performance data as well as treatment or prescription data have resulted in availability of an enormous amount of stored information. All this information is gathered in different databases, each with their own history, owner and administrator, design and purpose of data collection, quality criteria etc. This technological transformation has rarely been a coordinated process, but rather represents different initiatives taken by the veterinary authorities, by reference laboratories, public health authorities, private stakeholders, academia, and more generally, by the different ministries in the government that has developed administrative databases. The technological transformation opens new possibilities and the potential of these possibilities is not yet clearly described or understood.

The complexity of many global challenges on animal health and related issues requires that all these data are integrated to a higher extent than is done today to improve effectiveness in surveillance and control of health issues, including risk assessment and development of guidance for best practice. Integration does not necessarily mean that data from different sources are directly merged, but merely that the information from different sources is used in a coordinated effort to address complex research questions or challenges from veterinary- and human public health, including the massive challenges related to climate change and antimicrobial drug resistance. An integration of data requires that the databases can fulfill a number of demands including documentation of data sources, data flow, database structure and control procedures to ensure data quality and security. As an example, the European Food Safety Authority (EFSA) regularly collects data from the member states of the European Union (EU), and these data need to be harmonized to increase data quality and reduce biases/uncertainties in risk assessments (1). Moreover, the general need for guiding principles for scientific data management and stewardship has been emphasized (2). There are multiple examples that integration of health information across species is beneficial in terms of identifying emerging health issues (3), understanding risk factors and transmission mechanisms (4, 5) and controlling health issues (6), but as mentioned above we argue that the use of data can and should be improved considerable in the future in order to address emerging threats in a much more timely and effective way.

The objective of this paper was to present the different One Health data types available in Denmark, including data on animal demographics and movements, animal, and human medicine prescription, diagnostic test results and other health related data sources including relevant data sources on human patients and demographics. Furthermore, we mention some administrative databases that have proven to be of value for research and risk assessment. Another objective was to suggest approaches for better integration and improved use of data to provide evidence for risk-based policies to protect animal and human health. This will be discussed in the light of data ownership and requirements due to the European General data Protection Regulation (GDPR). To be more specific, the paper focuses on major production animal species and zoonotic agents, which will be exemplified by three illustrative cases:

1. The Danish Integrated Antimicrobial Resistance Monitoring and Research Programme (DANMAP)
2. *Salmonella* Dublin
3. *Campylobacter* infections.

## Methods

### Identification of Existing Databases and Their Documentation of Content

Databases with potential relevance for animal health were identified based on the research needs in the veterinary contingency work. In addition, the Danish legislation was scrutinized to identify data that must be recorded according to legislative orders. Furthermore, the authors have for many years been involved in research projects using databases and therefore have knowledge about many additional databases. Those research activities have often involved direct engagement of database owners and administrators who could provide additional details on the content of databases as well as the origin and flow of data.

In addition to databases directly related to animal health, databases in human health that can be related to occurrence of specific pathogens in livestock were included. As examples, we present research and surveillance on the occurrence of antimicrobial resistance, *Salmonella* Dublin and *Campylobacter* infections.

The databases were examined in relation to ownership, intended purposes of the database, target and study populations, metrics and information used, measuring methods (observers, diagnostic tests), recording procedures, data flow, database structure, and control procedures to ensure data quality.

### Context—Setting and Population

Denmark is a Scandinavian country of 43,000 km^2^, a population of 5.8 million (2018) and a life expectancy at birth of 80.9 years (7). The health system is tax-funded and visits to general practitioners and hospital admission are free of cost to all residents. Secondary health institutions are administered at regional level. A number of administrative registers are maintained all using a common key (the civil registry number, which is a unique code, provided to all individuals with residence in Denmark). Access may be given to linked, anonymized information from such registers for research purposes, and thereby the Danish population has been described as “one big cohort” (8).

The largest livestock sector is the pig industry. There are around 3,000 pig farms in Denmark with more than 12 million pigs on farm and producing more than 17 million pigs annually sent for slaughter in Denmark at a few large cooperative abattoirs, and around 14 million weaned pigs sold for export, mainly to Germany (9, 10). Around 90% of the produced pork is exported leading to a high demand for data used for breeding, quality, food safety, animal welfare, and traceability purposes. Therefore, the industry hosts and manages several databases for documentation purposes. The second largest livestock industry is the cattle sector with 1.5 million cattle including around 560,000 dairy cows in ~2,800 dairy farms producing milk and milk products for a few dairy companies that export to a large market. Denmark also has around 180 broiler chicken farms that produce around 114,000,000 broilers for slaughter every year (11). All livestock holdings are identified in a central registry, which will be discussed below. This enables data to be linkable. Furthermore, it is possible to integrate the human health and veterinary databases by e.g., geographic coordinates or postal codes.

## Details to Understand Key Programmatic Elements

A key element of infrastructures in both monitoring and surveillance programs are the existence of high quality databases. A full list of identified databases and their variables are outlined in Tables 1–5. In Supplementary Material, some key features from selected databases are presented as background information to understand the potentials in the cases used for exemplification.

**Table 1:** Databases on animal health embedded in Danish legislation with information publicly available.

**Database name**	**Purpose**	**Variables**
CHR Herd level	Demographic information on herd level; population composition	Owner; geographic location; species; herd size; notifiable diseases
CHR Animal level Cattle	Demographic information on animal level	Birth date; birth condition; sex; movement (both sender and receiver); date of slaughter; date of death
Movement database (pigs)	Veterinary preparedness	Animal type, no transported, mortality, date; Source and destination herds Trucks incl. nationality
VetStat	Recording of prescription medication at herd level	Medication name and active substance; species; age group; ordination group; Vet authorization and practice no; Drug store ID; prescription date etc.
Meat inspection recordings	Food safety; price deduction for farmer	Abattoir ID; animal category; clinical findings at ante-mortem inspection; pathological lesions observed post-mortem
Zoonosis register	Surveillance of Salmonella	Antibodies in meat juice and blood
Welfare control data	Recording of Danish Veterinary and Food Administration control data	Reason for control; visit date No of infringements of animal welfare legislation: Warning, enforcement notice or police report

**Table 2:** Databases on animal health in Denmark at national laboratories.

**Database name**	**Purpose**	**Variables**
SSI LIMS	Diagnostics and surveillance	Notifiable infectious diseases, date species, pathogens etc.
Ester/DTU-VET Center for diagnostik	Diagnostics and surveillance	Endemic diseases, date, species, pathogens etc.

**Table 3:** Databases in Denmark owned by the industry or privately owned.

**Database name**	**Purpose**	**Examples of variables**
Cattle database	Breeding, health advice, research	Milk yield, disease treatments, reproduction, Salmonella Dublin surveillance levels and diagnostic results from individual cattle and bulk-tank milk
SPF-database	Secure good biosecurity/health status; Document freedom	Disease status on several well defined contagious diseases
Efficiency control pigs	Production management	Number of weaned pigs, feed efficiency, growth
Poultry‘Kik’ data	Quality control in chicken production	GIS-coordinates, distance to neighbors, buildings, hygiene, climate, mortality
Efficiency-control poultry	Production management	Growth, egg production, feed consumption, mortality

**Table 4:** Selected databases in Denmark on human health that can be related to animal health or other animal related information.

**Database name**	**Purpose**	**Variables**
Register of enteric infections	First-positive (6 month period) patients of diagnosed gastrointestinal bacterial infections	Patient identifier (cpr number), date of sample, requesting doctor, laboratory, bacterial diagnosis (species level), foreign travel
Clinical disease notification database	Notified patients with a series of specified infectious diseases	Patient identifier, diagnosis, dates, relevant clinical information (underlying illness etc.), mode of transmission, place of infections, disease specific characteristics
Danish Microbiology Database (MiBa)	Information of clinical microbiological analyses performed since 2010	Patient identifier, diagnosis, dates, requesting doctor, laboratory—data are unstructured upon receival.
The National Patient Register	Hospitalized patients	Patient identifier, diagnoses codes (ICD8/10), in/out dates, examinations and treatment information, and more.
The Danish Cancer Register	All cancer patients	Patient identifier, relevant dates, type and location of cancer, treatment information, and more.
Euro-MoMo	Mortality data from Denmark and other European countries	Data of death per age group from Denmark and 20 other countries/states.

**Table 5:** Selected administrative databases in Denmark with relevance for One Health surveillance or research.

**Database name**	**Purpose**	**Variables**
Danish Civil Registration System	Population register	Person identifier (cpr number), first-level family members, current and previous addresses, vital status, civic status, and more.
Register of Causes of Death	Medical post-mortem examination registrations	Person identifier and time, cause, place, and manner of death.
Central Register of Buildings	Buildings register	Address, purpose, size, installations, construction data, dates of construction, and amendments.
Meteorological data	Weather and climate data	Area and period, temperature, wind, rainfall, and more.

In the following, further details on the data and their potentials are provided in three illustrative cases of monitoring health and disease in Denmark.

### Illustrative Cases

#### The Danish Integrated Antimicrobial Resistance Monitoring and Research Programme (DANMAP)

##### Background

DANMAP collects data from several different sources including food and hospital laboratories, slaughter plants, veterinary practices as well as general practices for people. DANMAP functions as a surveillance system of both the consumption of antimicrobial agents as well as the occurrence of resistant bacteria in the three sectors: livestock, food, and humans (12, 13).

##### Problem to be addressed

DANMAP was among the first examples of integrated surveillance for antimicrobial drug resistance, where integration was implemented in two dimensions: Human and animal, and drug use and resistance in both clinical infections and indicator bacteria. Thereby, DANMAP has served as a source of inspiration for many other countries (14). However, the concept of DANMAP has, by and large, remained constant over the years (15) with a published report available often in the fall of the coming year, thereby creating a time lag of 9 to 11 months before annual data become available. Hence, an important objective is to make the data available in real time, and also to continuously improve use of the data sources beyond descriptive analyses.

##### Data availability

DANMAP has from the beginning taken advantage of the databases on antimicrobial drug use held by the Danish Medicines Agency (Table 1), whereas data on antimicrobial sensitivity testing has been available only after long delays. This is partly due to a work intensive procedure, where data are collected from different microbiological laboratories that use different data formats. Hence, a lot of resources have been used to import, merge and clean the data before data analysis can begin. Furthermore, clinical data have been meager, which prevents analysis of risk factors for drug resistance. Denmark is now working on an online access to human resistance testing (16), and the report for 2018 will be a prototype for this, and we hope that veterinary data also will be available soon.

##### Potentials

With online availability of data, the collation and analysis of data can be developed into an on-going activity that improves the timelines and enables rapid identification of emerging trends (15). Furthermore, richer data including risk factors and clinical outcomes will enable risk assessment and serve as a tool for research. The full potential of this transformation is not yet fully described, and will depend on legal issues as well as the resources that will be available for data analyses and data visualization.

A recent FAO report (17) states under lessons learned that “Change takes time. Most of the initiatives have been implemented gradually, giving farmers and veterinarians time to adjust and devise smart solutions,” “The well-organized Danish agricultural industry has been an important factor in achieving this success,” and “The proposed solutions may not be directly transferable to other countries as they may have different incentives to drive change at all levels of society.”

Such statements underlines the need for activities at the community level to achieve the potentials.

#### Salmonella Dublin

##### Background

In Denmark, there is an on-going surveillance and control program for *Salmonella* Dublin in cattle. Although, *S*. Dublin relatively seldom occurs in humans, it is associated with a high case fatality rate and it is therefore considered as an important zoonosis (18, 19). In Denmark, *S*. Dublin is notifiable meaning that owners of animals with a suspicion of salmonellosis must call a veterinarian who should seek to confirm or reject the diagnosis (20), and laboratories that isolate salmonella bacteria have to report the results to the veterinary authorities.

##### Problem to be addressed

Hitherto, no direct association between human cases and *S*. Dublin in cattle has been demonstrated in the literature (19). However, work on comparison of whole-genome sequencing of strains from both populations is currently on-going. If the occurrence of *S*. Dublin in cattle and humans is correlated, the information on occurrence and location of infected premises and infected cattle can be used to assess the risk of transmission from cattle to humans through contact. Furthermore, it can inform policies on how to prevent transmission from live animals or transmission via contaminated meat.

##### Data availability

The legislation requires that all farms are categorized into one of three defined infection levels. Monitoring of *S*. Dublin is mainly based on bulk tank milk from dairy herds and blood samples from non-dairy herds (20). The bulk tank samples are obtained repeatedly with 3 months intervals, and blood samples are collected either at the slaughterhouse when the herd delivers animals to slaughter or on-farm on the initiative of the farmer. The samples are analyzed for antibodies directed against *S*. Dublin (21). Level 1 herds are considered most likely free of *S*. Dublin. Level 2 is given to herds if (a) the herd does not live up to the Level 1 antibody test-criteria, (b) the infection status is unknown (e.g., insufficiently tested), (c) *S*. Dublin bacteria are detected or (d) there has been contact to cattle from a Level 2 or 3 herd. Finally, Level 3 is given to herds with (a) *S*. Dublin bacteria detection in a persistently infected Level 2 herd during mandatory intensified fecal sampling, or (b) diagnosis of salmonellosis (i.e., clinical disease). Level 3 herds are placed under official veterinary supervision with special hygienic restrictions including hygienic slaughter procedures for food safety reasons. The infection status of each herd is publicly available of the internet page of The Danish Food and Veterinary Administration (https://chr.fvst.dk). The surveillance scheme provides a large amount of longitudinal and repeated cross sectional data from all cattle herds in Denmark starting in 2002, which has been used frequently over the years for research and program development purposes (21–23).

##### Potentials

Previously, the correlation between the residence of human *S*. Dublin cases and distance to cattle farms has been investigated (19) concluding that the infection risk was independent of living near to cattle farms. However, the infection statuses of the cattle farms were not taken into account in that study. Hence, it could strengthen the investigation, if the *S*. Dublin status of the cattle farms at the time of the identification of the human case had also been included in the analysis. This is potentially possible as all necessary data are available and if data from different sources can be and are allowed to be integrated.

The process of furthering knowledge and better implementing data has been done in the Danish livestock and public health sector separately. The Danish cattle advisory center, SEGES, has a website with weekly updated status of national and regional prevalences and locations of test-positive cattle herds. SSI has an interactive webpage of laboratory results in which it is possible to get summary statistics of human *S*. Dublin cases including some demographics of these (see https://statistik.ssi.dk//sygdomsdata#!/?sygdomskode=SALM&stype=9&xaxis=Aar&show=Graph&datatype=Laboratory). Data are not integrated across species in these websites, and it is not known whether it would improve the control efforts in the surveillance and eradication programme to illustrate all of the data more clearly in both places. However, these two sources are used frequently by the working groups supporting decision making in the S. Dublin programme.

#### Campylobacter Infections

##### Background

Human illnesses caused by foodborne and zoonotic infections constitute important public health problems in modern developed societies. A good understanding of the infection sources, risk factors, and the disease burden is necessary in order to be able to devise evidence-based ways of addressing these challenges. This effort demands a cross-sectorial approach and combining data sources collected from different sectors is valuable or even necessary. To illustrate this, we will here briefly mention research, where different Danish data sources have been combined in order to address One Health research questions, using *Campylobacter* infections as an example.

##### Problem to be addressed

*Campylobacter* is the leading cause of gastrointestinal infections in Denmark, as indeed in Europe as a whole (24). In Denmark, several successive national action plans have aimed at reducing the number of infections in poultry and humans (25). This has potentiated the need to address the possible transmission routes, which has been done via linkage of data from different sources.

##### Data availability

Data from the national monitoring of poultry flocks at slaughter were combined with the national surveillance data on human patients. Climate data (in particular historic temperature and rainfall series), geographical data including geo-coded address data, national data on buildings, data from the population register (to form cohort and control reference populations), and data from the National Patient Register (Tables 4, 5) were extracted and linked. Population-based case-control studies have established consumption of fresh chicken meat (26) and a number of other factors including pets and leisure activities (27) as major risk factors for human infections. Locating human cases addresses, geocoding them and performing register-linkage to housing information showed risk of infection to be pronounced in rural areas in particular among children (28). Analyzing meteorological data showed both chicken flock infection levels and human infections to correlate with increasing temperature in Scandinavia (29) and, at a more detailed level, in Denmark (30). Linking human surveillance data with several social information registers, showed *Campylobacter* to primarily affect the affluent segments of society (31).

##### Potentials

Combining database information for the purpose of research into transmission routes of *Campylobacter* has shown to have a significant potential and such studies should be further pursued. On-going studies at the SSI aim to model the effect of heavy rain-events for local outbreaks, model the effect of different climate change scenarios in Scandinavia and describe the burden of infections by following post-infectious sequelae of patients and non-patients using the population registry and hospital diagnosis data.

## Discussion and Conclusions

The globalization with increasing traveling and movements of animals and their products challenge an efficient food production providing global food security and food safety. Further, climate change triggers changes in spread of disease demanding increased preparedness in our disease surveillance and control. The developments in antimicrobial resistance are mentioned as one of the most important global health threats by WHO. All these developments require that both the veterinary and public health authorities are provided with the most precise and up to date evidence for disease occurrence and spread in order to take effective and innovative decisions on interventions. In order for this to happen, we must utilize our technical capacities to provide access to information and databases across veterinary fields, food products, human health, and administration. In this paper, we have demonstrated that we have reached important milestones in these efforts. For example, the Danish *Salmonella* Dublin eradication programme was initiated as a joint initiative between the veterinary authorities and the Danish cattle sector in 2002 based on research and documentation activities over the previous 5 years. The programme has been led by a cross-sectoral steering committee and supported by a technical working group with representatives from all essential stakeholders over the years including industry, laboratory, academic, and public health/food safety institutions. This approach builds on experiences from prior successful disease control programmes in the cattle sector (32), but still there is a much greater potential that is not yet utilized.

The campylobacter examples show how human and veterinary health data can be linked with databases not normally used for public health, such as the databases on climate or housing, leading to new insights in the epidemiology of these infections. Furthermore, they emphasize that use of national registers, including the population register, facilitates analyses using the entire Danish population as a cohort, thereby giving considerable statistical power.

We recommend that individual countries meet the mentioned requests by collaboration between sectors and authorities. In particular, data must be made available and there is a need to fund development projects with the purpose of facilitating data sharing and providing platforms for data analysis and visualization. The process of furthering knowledge and implement better use of data must include elaboration of systematic database protocols and transparent visualization of data including descriptive analysis of key variables (disease occurrence, medicine consumption etc.), which will enable the next step of integrating the data. Such a systematic approach will increase the generalizability of the findings of the three illustrative cases to include databases on climate, vectors, and other information related to animal and human health. The increased availability and transparency of databases and their specific data can to a much higher extent be used at university both in teaching specific courses, and when the students need to write bachelor or master theses. Further, the research based advisory activity for the authorities can use the data to improve monitoring of the current situation and hence set up better warning systems both at the national level, and also at herd level for use by the local advisors and veterinary practitioners. Finally, researchers can elaborate new and more innovative research questions involving several animal species and humans.

Special attention should be given to secure that rules of GDPR will not prevent an efficient use of data. Concerning GDPR, it is stated in the legislation that data must be collected explicitly for given purposes and that data collection shall not include more information than is needed for the given purposes. In addition, there are regulations to secure the anonymity of individual persons. Such requirements will sometimes make it difficult to integrate data on the levels of individual people or individual farms and it may be necessary to integrate data at a higher level, e.g., municipality or region. Increased use of professional data managers can in future be a very important step in helping researchers and other users of data to overcome obstacles concerning GDPR. Further limitations can consist of unwillingness to provide data on matters that may be sensitive for industry and authorities. Also lack of experience with use of data can be a problem. Professional data managers can in general also ensure that the quality criteria for use of databases being put forward in many guidelines (e.g., 1, 2) are met. For data integration to be feasible such professionals should work across different sectors to secure coherence in the ontology of the databases. If, for example, different databases use terms such as “diagnosis” or “disease” they need to be defined in the same way or at least it must be very transparent how the terms are defined (and scored/graded) in the different databases (33, 34). In addition, the use of definitions and coding may change within the same database over time and such changes must be fully transparent for the user.

Challenges have previously been identified to One Health surveillance (e.g., legal and data sharing issues, unclear responsibilities and structural barriers between ministries) (35). Such challenges continue to exist and it takes considerable effort from several parties to overcome these challenges. As illustrated by the three cases presented in this paper, it is very important continuously and actively to support the use and integration of data.

## Author Contributions

All authors listed have made a substantial, direct and intellectual contribution to the work, and approved it for publication.

### Conflict of Interest Statement

The authors declare that the research was conducted in the absence of any commercial or financial relationships that could be construed as a potential conflict of interest.
