# Supplementary material for: Opportunities for Improved Disease Surveillance and Control by Use of Integrated Data on Animal and Human Health
Source: Front Vet Sci. 2019 Sep 13;6:301. doi: 10.3389/fvets.2019.00301 (PMC6753377; doi:10.3389/fvets.2019.00301)
Supplement: Supplementary file 1 [file Data_Sheet_1.docx]

**Supplementary material**

**Description of relevant databases related to animal and human health in Denmark**

*Description of relevant databases concerning animals*

A cornerstone in veterinary contingency planning is the identification and tracing of animals. Further, traceability is important for identification of sources for food safety problems. This is secured in the Danish order of registration of herds in the Central Herd Registry (CHR) (BEK 1408, 2016; LBK 925, 2017). Thus, every herd must be registered in CHR, where the CHR-number provides identification of the geographic location of the herd (Table 1). For the different animal species, more details are given (BEK 598, 2017). For example, each individual cattle must be physically ear-tagged with a unique identification number. Furthermore, the date of birth and most movements, as well as the date of death or slaughter must be recorded (Table 1). For movements, exact information on both sender and receiver must be recorded. All this information is also available in a public database (<http://chr.fvst.dk>). For pigs, a tattoo with the herd identification number is sometimes sufficient when the animal is moved for slaughter, but the individual pig can always be traced back to its herd of origin. Importantly, the CHR also contains information on occurrence of notifiable diseases, availability of food chain information, *Salmonella* Dublin classification of the herd and whether the herd is allowed to sell animals or move animals off farm.

The Danish Medicine Agency (“Lægemiddelstyrelsen”) controls the production and distribution of medicinal products. Due to the increasing occurrence of antimicrobial resistance, it was decided that all medication prescribed for production animals must be reported to the public register “VetStat” (BEK 1647, 2018). The use, supply or prescriptions are reported by the herd owner, the herd veterinarian, the feed mill or the pharmacy. The recording must among other things include information on the medication name and active substance, animal species, age group, ordination group and the amount of medication (Table 1). The specific purposes of VetStat are to have an efficient tool to monitor use of veterinary drugs in animal production, to help practitioners in their work as herd advisors, to reveal inappropriate or illegal use of antibiotics and to provide possibilities for researchers to examine the association between antibiotic use and the occurrence of antimicrobial resistance (Dupont et al., 2017).

The so-called “zoonosis directive” in the European Union states that: “Member States shall ensure that data on the occurrence of zoonoses and zoonotic agents and antimicrobial resistance related thereto are collected, analysed and published…” (Directive, 2003). Thus, zoonotic infections in both humans and animals are monitored, and the monitoring also includes the occurrence of zoonotic agents in food and feedstuffs. As one example, meat juice samples from slaughter pigs are collected at slaughterhouses and examined for antibodies directed against salmonella (BEK 1426, 2018).

*Description of relevant databases concerning humans*

A number of registries hold information of Danish residents and human patients. Danish health registers are maintained by the Danish Health Data Authority (<https://sundhedsdatastyrelsen.dk>) or Statens Serum Institut (SSI: [www.SSI.dk](http://www.SSI.dk)) under the Ministry of Health. Information about national administrative registers and data access may be found through Statistics Denmark (https://www.dst.dk/en).

Important human health registries/databases are listed in Table 4. Disease surveillance data are collected as part of the Danish mandatory disease notification systems. They are regulated through the Statutory Order on Physicians' Notification of Infectious Diseases by the Danish Health Authority and managed by the Statens Serum Institut (<https://en.ssi.dk/surveillance-and-preparedness/surveillance-in-denmark/mandatory-notification-systems>). Surveillance consists of diseases reportable either by the clinical laboratories or directly by clinicians and supplemented with a number of voluntary systems. Case based surveillance of zoonotic diseases is primarily performed through the laboratory system. Thus, person-identifiable information (without clinical details) on gastrointestinal bacterial infections are maintained in Register of Enteric Infections (Simonsen et al., 2008). Individually (clinical) notifiable diseases within the One Health area include *Shigella*, methicillin-resistant *Staphylococcus aureus* (MRSA), shiga-toxin producing *Escherichia coli* and haemolytic uremic syndrome and suspected food- or waterborne outbreaks. Since 2010, a common database collecting all analyses performed in clinical microbiological laboratories in Denmark (negative and positive) have existed. This database, the Danish Microbiology Database (MiBa), is an important resource in disease surveillance (Voldstedlund et al., 2014) and the strategy for the future development of the Danish disease surveillance involves automatic case capture and definition based on MiBa data.

The National Patient Register contains information on Danish admitted patients to all somatic hospital departments from 1977 and onwards, and since 1995 also to ambulatory, emergency and psychiatric departments (Schmidt et al., 2015). The main purpose of the register is quality control and the reimbursement of cost of procedures and treatments. However, in a One Health perspective, relevant patients may also be identified using this register and it may serve as an important means to evaluate underlying illness or sequelae in specific (patient) groups within Danish society as well as associations between illnesses and distance to potential environmental risk or protective factors such as livestock farms.

Table 5 lists non-health registries of importance to One Health. This list only represents a small subset of many registers with potentially useful information. All Danish residents are listed in CPR, the Central Population Registry (Danish Civil Registration System) by a unique number given on registration of birth or immigration (Pedersen et al., 2006). This number, the cpr number, is used as key identifier in other registries, including the health registries mentioned in Table 4. CPR was established in 1968 and has public administration as its main purpose. The Central Register of Buildings and Dwellings (BBR) is a national register containing information originally collected for administrative purposes at the municipal level of all buildings in the area. It lists data on all properties in Denmark, residential, commercial or public. The Danish Meteorological Institute collects climate and weather data, which may be of importance in a One Health perspective. Data from these databases are expected to be public domain data from 2022.

**References**

BEK 1408, November 29 (2016) Government order of registration of herds in CHR (In Danish)

BEK 598, May 31 (2017) Government order on registration and movement of animals (In Danish)

BEK 1647, December 18 (2018). Government order on the use of medication (In Danish)

BEK 1426, November 30 (2018) Government order on Salmonella in swine (In Danish)

DIRECTIVE 2003/99/EC OF THE EUROPEAN PARLIAMENT AND OF THE COUNCIL of 17 November (2003) on the monitoring of zoonoses and zoonotic agents, amending Council Decision 90/424/EEC and repealing Council Directive 92/117/EEC

Dupont, N., Fertner, M., Birkegaard, A. C., Andersen, V. D., Nielsen, G. B., Kruse, A. B., & Knegt, L. V. D. (2017). Improving institutional memory on challenges and methods for estimation of pig herd antimicrobial exposure based on data from the Danish Veterinary Medicines Statistics Program (VetStat). Available online at:

<https://static-curis.ku.dk/portal/files/199126055/1705.08663.pdf>

LBK 925, July 3 (2017). Government order on keeping animals (In Danish)

Pedersen CB, Gøtzsche H, Møller JO, Mortensen PB. The Danish civil registration system. A cohort of eight million persons. *Dan. Med. Bull.* (2006) 53: 441-449

Schmidt M, Schmidt SAJ, Sandegaard JL, Ehrenstein V, Pedersen L, Sørensen HT. The Danish National Patient Registry: a review of content, data quality, and research potential. *Clinical Epidemiology* (2015) 7: 449-490
